# Direct Contact with Endoderm-Like Cells Efficiently Induces Cardiac Progenitors from Mouse and Human Pluripotent Stem Cells

**DOI:** 10.1371/journal.pone.0046413

**Published:** 2012-10-02

**Authors:** Hideki Uosaki, Peter Andersen, Lincoln T. Shenje, Laviel Fernandez, Sofie Lindgren Christiansen, Chulan Kwon

**Affiliations:** 1 Division of Cardiology, Johns Hopkins University School of Medicine, Baltimore, Maryland, United States of America; 2 Laboratory of Reproductive Biology, University Hospital of Copenhagen, Copenhagen, Denmark; National University of Singapore, Singapore

## Abstract

**Rationale:**

Pluripotent stem cell–derived cardiac progenitor cells (CPCs) have emerged as a powerful tool to study cardiogenesis in vitro and a potential cell source for cardiac regenerative medicine. However, available methods to induce CPCs are not efficient or require high-cost cytokines with extensive optimization due to cell line variations.

**Objective:**

Based on our in-vivo observation that early endodermal cells maintain contact with nascent pre-cardiac mesoderm, we hypothesized that direct physical contact with endoderm promotes induction of CPCs from pluripotent cells.

**Method and Result:**

To test the hypothesis, we cocultured mouse embryonic stem (ES) cells with the endodermal cell line End2 by co-aggregation or End2-conditioned medium. Co-aggregation resulted in strong induction of Flk1^+^ PDGFRa^+^ CPCs in a dose-dependent manner, but the conditioned medium did not, indicating that direct contact is necessary for this process. To determine if direct contact with End2 cells also promotes the induction of committed cardiac progenitors, we utilized several mouse ES and induced pluripotent (iPS) cell lines expressing fluorescent proteins under regulation of the CPC lineage markers Nkx2.5 or Isl1. In agreement with earlier data, co-aggregation with End2 cells potently induces both Nkx2.5^+^ and Isl1^+^ CPCs, leading to a sheet of beating cardiomyocytes. Furthermore, co-aggregation with End2 cells greatly promotes the induction of KDR^+^ PDGFRa^+^ CPCs from human ES cells.

**Conclusions:**

Our co-aggregation method provides an efficient, simple and cost-effective way to induce CPCs from mouse and human pluripotent cells.

## Introduction

The availability of embryonic stem (ES) cells and induced pluripotent stem (iPS) cells has opened new fields of research and medicine[1]–[3]. ES/iPS cells have the potential to become all types of somatic cells, and therefore, they are potential cell sources for disease modeling, drug discovery and regenerative medicine.

Heart malformation is the most frequent form of human birth defects, and heart disease is the number one killer of adults worldwide [4]. The limited regenerative capacity of the heart is a major factor in the morbidity and mortality. Recent advances point to the potential of therapies based on cardiac progenitor cells (CPCs). CPCs can be purified from ES/iPS cells and manipulated in culture to expand and differentiate into various types of cardiac cells including cardiomyocytes, vascular endothelial cells and smooth muscle cells [5], [6]. Therefore CPCs might be potential cell types for cardiac regenerative therapy.

In recent years, several methods have been developed to induce CPCs from ES/iPS cells, based on defined culture media and temporal addition of specific concentrations of cytokines such as Activin A and BMP4 (Bone morphogenic protein 4) [7], [8]. However, due to cell line variations, individual ES/iPS cell lines require substantial optimization for efficient CPC induction [9], [10].

In contrast to variations in differentiation propensity in vitro, cell induction and differentiation are tightly regulated in vivo and often, neighboring cells play important roles for the cellular events through short-range signals. Based on our observation that early endodermal cells maintain contact with nascent pre-cardiac mesoderm in developing embryos, we determined if direct physical contact with endoderm promotes the induction of CPCs from pluripotent cells. Here, we report a novel method to induce CPCs from mouse and human ES/iPS cells.

## Materials and Methods

### Embryonic Stem Cell Derivation and Cell Culture

Mouse ES*^Isl1-Cre; Rosa-YFP^* and ES*^Isl1-Cre; Rosa-RFP^* cells harboring *Isl1^Cre^; Rosa^YFP^* or *Rosa^RFP^* were derived on irradiated mouse embryonic fibroblasts (MEF) in knockout (KO) DMEM supplemented with 15% fetal bovine serum (FBS), 0.1 mM nonessential amino acids, 2 mM GlutaMAX (Invitogen), 0.1 mM sodium pyruvate (Invitogen), 0.1 mM 2-mercaptoethanol (Sigma-Aldrich), and 100 U/ml leukemia inhibitory factor (LIF, Millipore), 3 µM CHIR99021 and 1 µM PD0325901 by standard procedures.

Mouse iPS*^Isl1-Cre; Rosa-YFP^* cells were generated from mouse skin fibroblasts and a gift from K.L. Laugwitz (Technical University of Munich, Germany) [11]._Mouse ES*^Nkx2^.^5-GFP^* cells were generated from the mouse E14 ES cell line, carrying the RP11-88L12/NKX2-5-Emerald GFP and a gift from B.R. Conklin (Gladstone Institute of Cardiovascular Disease, UCSF, San Francisco) [12]. Mouse ES (mES) cells were propagated feeder-free on gelatin-coated cell culture plastic (BD) and maintained in an undifferentiated state in GMEM supplemented with 10% FBS, 0.1 mM nonessential amino acids (Invitrogen), 2 mM GlutaMAX (Invitrogen), 0.1 mM sodium pyruvate (Invitrogen), 0.1 mM 2-mercaptoethanol (Sigma-Aldrich), and 2000 U/ml leukemia inhibitory factor (LIF, Millipore). Mouse ES cells were passaged every 2–3 days with TrypLE Express (Invitrogen) with daily medium changes. Human ES cells (H9) were propagated feeder-free on matrigel-coated cell culture plastic (BD) and maintained in an undifferentiated state in MEF-conditioned KO DMEM supplemented with 20% KO serum replacement and 8 ng/ml human basic fibroblast growth factor (FGF) [10]. End2 cells were a kind gift from Christine Mummery (Leiden University Medical Centre, Leiden, Netherland) [13]. End2 cells were propagated in D-MEM/F-12 supplemented with 7.5% FBS and 2 mM GlutaMAX, passaged every 2–3 days with TrypLE Express and never allowed to reach confluence.

### CPC Induction and Differentiation

For co-culture experiments, ES/iPS cells and End2 cells were dissociated to single cells and plated on ultra-low attachment plastic surface (Corning) in IMDM/Ham-F12 (Cellgro) (3∶1) supplemented with N2, B27, penicillin/streptomycin, 2 mM GlutaMAX, 0.05% bovine serum albumin, 5 ng/ml L-ascorbic acid (Sigma-Aldrich), and α-monothioglycerol (Sigma-Aldrich) at final concentration of 75,000 cells/ml. Medium was changed after 3 days of co-culture. For human ES cells, Rock inhibitor (Y27632) was added to permit survival of cells in single cell suspension [14].

For cardiac differentiation, CPCs from co-cultures were purified by FACS and plated on gelatin-coated cell-culture plastic (BD) in StemPro34 (Invitrogen) supplemented with 10 ng/ml L-ascorbic acid, penicillin/streptomycin and 2 mM GlutaMAX.

### Flow Cytometry

ES cells were dissociated and percentages of Pdgfra-PE+ and Flk1-APC+ were analyzed using Accuri C6 Flowcytometer (BD Biosciences) and FlowJo software. For sorting, cells were dissociated and resuspended in 0.1% FBS/20 mM Hepes/1 mM EDTA/PBS without Ca^2+^ and Mg^2+^ and sorted on a FACSAria (BD Biosciences). The following antibodies were used: anti-Flk1 antibody conjugated with APC (eBioscience) and anti-Pdgfra conjugated with PE (eBioscience) for mouse ES/iPS cells, and anti-KDR antibody conjugated with APC (Miltenyi Biotec) and anti-PDGFRa conjugated with PE (R&D Systems) for human ES cells.

### Quantitative RT-PCR

RNA was extracted with TRIzol (Invitrogen). Reverse transcriptase–quantitative PCR (qPCR) was performed using Multiscribe reverse transcriptase (Applied Bioscience) and TaqMan probes on the ABI 7900HT (Applied Biosystems), according to the manufacturer's protocols. Optimized primers from TaqMan Gene Expression Array were used. Expression levels were normalized to Gapdh expression. All samples were run at least in triplicate. Real-time PCR data were normalized and standardized with SDS2.2 software.

### Histology and Immunohistochemistry

Embryo cryosections were stained with antibodies against Troma1 and RFP followed by secondary detection with appropriate Alexa Fluor conjugated antibodies. Immunostaining was performed on cultured cells that were fixed in 4% paraformaldehyde with primary antibodies against cardiac troponin T (cTnT), Vimentin or alpha smooth muscle actin (aSMA) followed by secondary detection with appropriate Alexa Fluor conjugated antibodies.

## Results

### Early Endoderm Is in Physical Contact with Nascent Cardiovascular Progenitors

**Figure 1:**
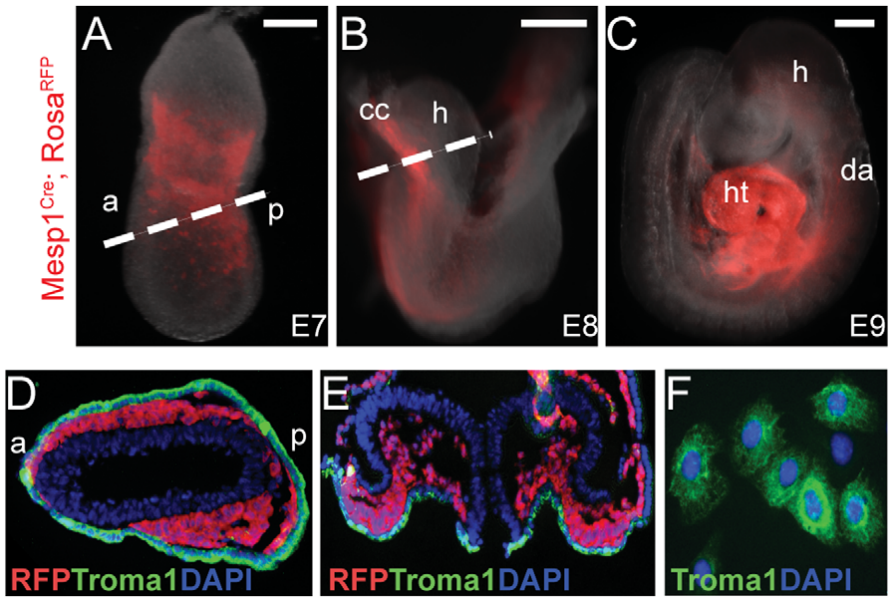
Early endoderm is in physical contact with nascent cardiovascular progenitors. **A–C**, *Mesp1^Cre^; Rosa^RFP^* embryos at E7 (A), E8 (B), and E9 (C). **D, E**, Transverse section of E7 (D) or E8 (E) *Mesp1^Cre^; Rosa^RFP^* embryo stained with RFP (red), Troma1 (green), and DAPI (blue). The cutting plane is indicated by a dotted line in (A) or (B). **F,** Endoderm-like (End2) cells stained with Troma1 and DAPI. DAPI was used to counterstain the nuclei. Scale bars, 75 (A), 200 (B), 250 (C) µm. a, anterior; p, posterior; cc, cardiac crescent; h, head; ht, heart; da, dorsal aorta.

The basic helix-loop-helix transcription factor Mesp1 is transiently expressed in the earliest cardiovascular progenitor cell population that gives rise to the entire heart and vasculature [15]. To trace the cardiovascular cell lineage during early development, we crossed *Mesp1^cre^* mice [15] with *Rosa^tdTomato^* mice [16], which leads to constitutive activation of red fluorescent protein (RFP) in Mesp1^+^ cells and their progeny. We obtained embryos from embryonic days (E)7–9. RFP^+^ cells appeared at lateral plate mesoderm at E7 (Figure 1A) and formed the cardiac crescent at E8 (Figure 1B). By E9 RFP^+^ cells contributed to the entire heart while some contributed to vasculature as reported (Figure 1C) [15]. We found that RFP^+^ cells maintained close contact with the endodermal layer, marked by expression of the endodermal gene Troma1 [17], throughout the early embryogenesis (Figure 1D, E). Based on this observation, we hypothesized that physical contact with early endodermal cells positively affects the induction of pre-cardiac mesoderm from pluripotent cells. To test this hypothesis, we utilized endoderm-like (End2) cells, which were established from embryonic carcinoma cells [13] for the following reasons: (1) End2 cells were shown to enhance cardiomyogenesis of human ES (hES) cells [13], (2) They are easy to culture and maintain in vitro, (3) They express high levels of Troma1 (Figure 1F).

### Co-Aggregation with End2 Cells Efficiently Induce Early Cardiac Progenitor Cells from Mouse Embryonic Stem Cells

**Figure 2:**
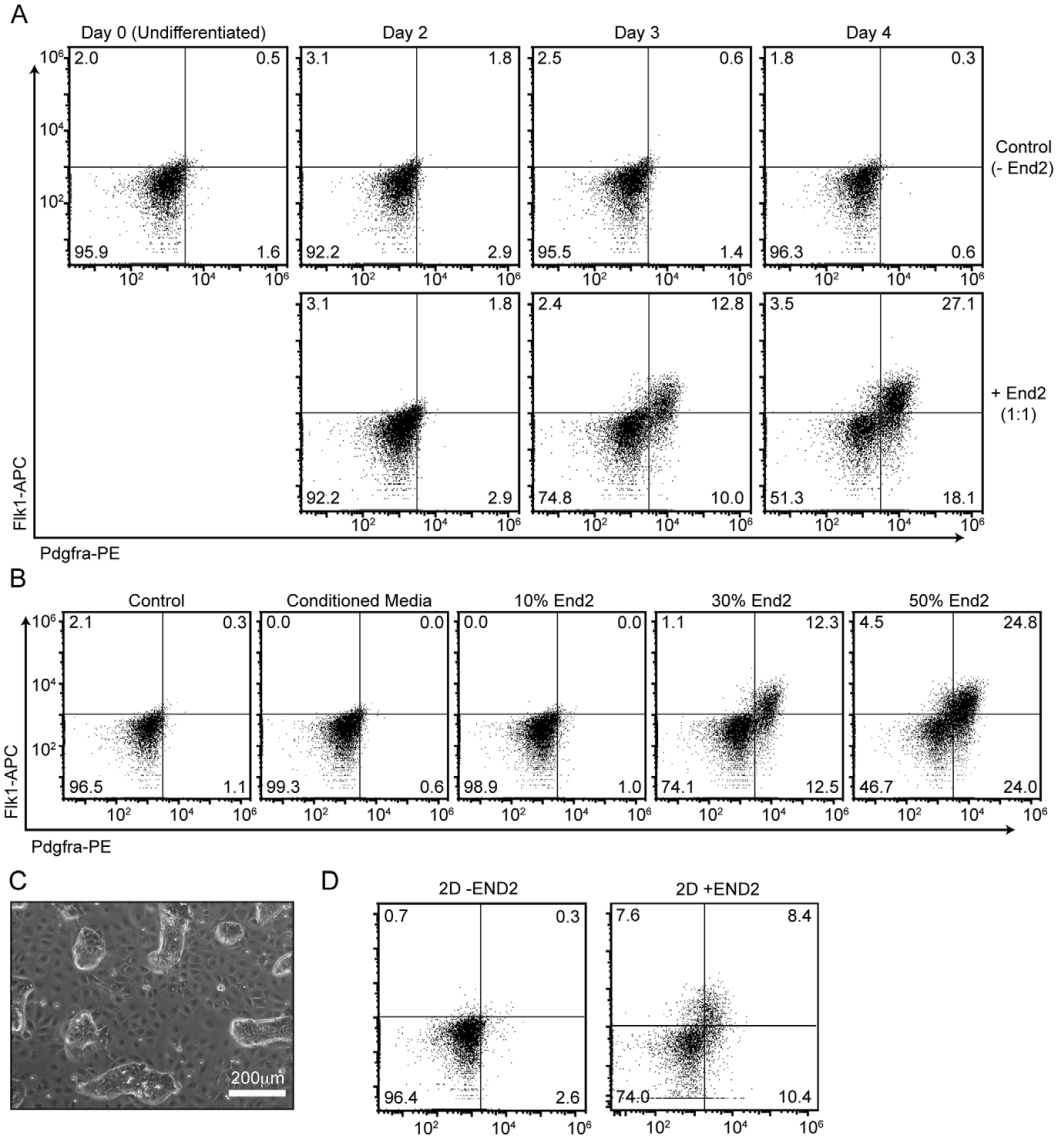
Co-aggregation with endoderm-like (End2) cells potently induces early cardiac progenitor cells from mouse ES cells. **A,** Time course of early CPC markers, Flk1 and Pdgfra, during mouse ES cell differentiation with and without End2 cells. **B,** Expression profiles of Flk1 and Pdgfra. End2 cells promote CPC induction in a dose-dependent manner. **C,** mES cells colonies formed in 2D co-culture with End2 cells. **D,** Expression profiles of Flk1 and Pdgfra. Early CPCs were not efficiently induced in 2D co-culture.

To determine if physical contact with End2 cells affect the induction of pre-cardiac mesoderm from pluripotent cells, we differentiated mES cells by co-aggregation with End2 cells in serum-free media and monitored the emergence of the early CPC pre-cardiac mesoderm markers, Flk1 and Pdgfra, by flow cytometry [18]. The co-aggregation resulted in a marked increase in the number of Flk1^+^ Pdgfra^+^ cells from day 3. The number of Flk1^+^ Pdgfra^+^ cells peaked at day 4. However, Flk1^+^ and/or Pdgfra^+^ cells were barely detectable in control mES cells (Figure 2A). The induction of the early CPCs was dependent on End2 cell doses with a 1∶1 co-aggregation showing the strongest activity (Figure 2B).

To determine if secreted factors of End2 cells are sufficient to induce Flk1^+^ Pdgfra^+^ cells, we differentiated mES cells with End2-conditioned medium. However, End2-conditioned medium did not affect the CPC induction (Figure 2B). To further examine the role of End2 cells, we cocultured mES cells and End2 cells as a monolayer. Similar to ES cells grown on feeders in the presence of LIF, ES cells formed distinct colonies surrounded by End2 cells, with only the outer boundary of ES colony making direct contact with End2 cells (Figure 2C). However, the appearance of Flk1^+^ Pdgfra^+^ cells were significantly reduced as compared to EB formation (Figure 2D), suggesting that the inductive effect by End2 cells is likely mediated through short-range signals in form of cell-cell contact.

### Co-Aggregation with End2 Cells Promotes Induction of Committed Cardiac Progenitor Cells from Mouse Pluripotent Stem Cells

**Figure 3:**
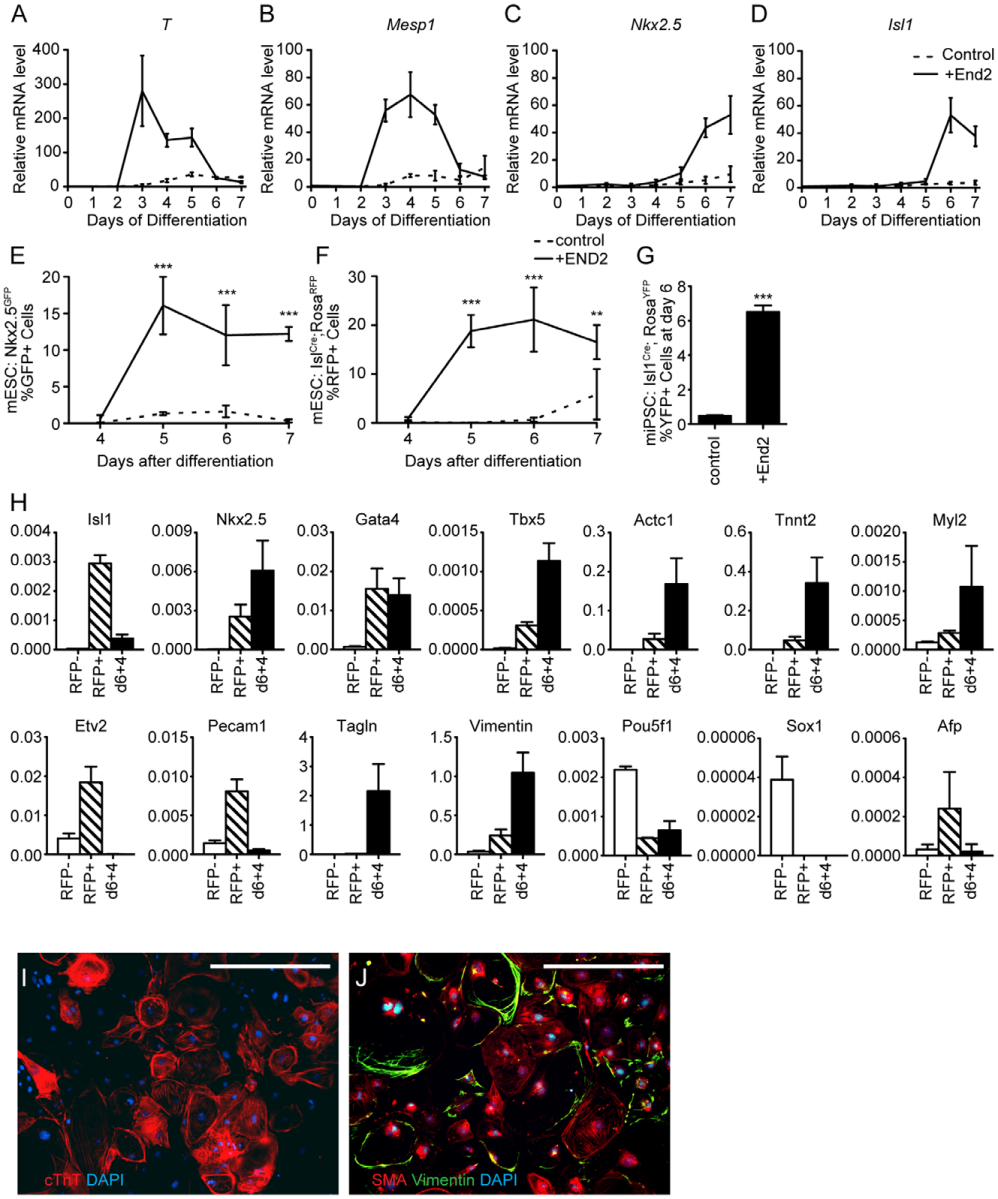
Co-aggregation with End2 cells efficiently induces Isl1^+^/Nkx2.5^+^ cardiac progenitors from mouse pluripotent cells. **A–D,** Gene expression profiles during ESC differentiation with and without End2 cells (n = 3, mean±SD). Mesendoderm marker, *T* (A), pre-cardiac mesoderm marker, *Mesp1* (B), cardiac progenitor cell/cardiomyocyte marker, *Nkx2.5* (C), cardiac progenitor cell marker, *Isl1* (D). **E,** Ratio of GFP^+^ cells for day 4–7 of differentiation from ES*^Nkx2^.^5-GFP^* cells. **F,** Ratio of RFP^+^ cells for day 4–7 of differentiation from ES*^Isl1-Cre; Rosa-RFP^* cells. **G,** Ratio of YFP^+^ cells for day 4–7 of differentiation from iPS*^Isl1-Cre; Rosa-YFP^* cells. (n = 3, mean±SD, **; p < 0.01, ***; p < 0.001, Two-way anova and Bonferroni post-tests (E–F) and unpaired t-test (G)) **H,** Gene expression profiles of RFP^+/−^ cells isolated at day 6 and RFP^+^ cells differentiated for additional 4 days after sorting (Day6+4), normalized to Gapdh. **I–J** immunostaining of cells at 4 days after sorting of YFP^+^ cells at day 6. Cardiac troponin T (red) and DAPI (blue) (I). Smooth Muscle Actin (SMA, red), Vimentin (green) and DAPI (blue) (J). Scale Bars, 400 µm.

While Flk1^+^ Pdgfra^+^ cells represent early cardiovascular progenitors, they are not fully committed to cardiac lineages and require additional exogenous signals to become committed CPCs, which are indicated by expression of the cardiac transcription factors Nkx2.5 or Isl1 [19], [20]. To investigate the role of End2 cells in CPC development, we examined several CPC markers by qPCR. The mesendodermal gene *brachyrury* (*T*) was transiently expressed and peaked at day 3 of co-culture (Figure 3A). The pre-cardiac mesodermal gene *Mesp1* started to express at day 3 and peaked at day 4 (Figure 3B). This is consistent with the expression of Flk1 and Pdgfra, analyzed by flow cytometry. The CPC genes *Nkx2.5* and *Isl1* were both expressed from day 6 (Figure 3CD), suggesting the emergence of committed CPCs. Without End2 cell aggregation, however, none of these genes was expressed (Figure 3A–D).

To test if co-aggregation with End2 cells promotes the induction of committed CPCs, we generated an mES (ES*^Isl1-Cre; Rosa-RFP^* and ES*^Isl1-Cre; Rosa-YFP^*) cell line that constitutively expresses red fluorescent protein (RFP) and yellow fluorescent protein (YFP) in Isl1^+^ cells, which predominantly give rise to the outflow tract and right ventricular cells [20]. We also utilized the ES*^Nkx2^.^5-GFP^* cells that express green fluorescent protein (GFP) from the *Nkx2.5* locus [12]. Nkx2.5^+^ progenitors predominantly give rise to the left ventricular cells [21]. We found that co-aggregating ES*^Nkx2^.^5-GFP^* or ES*^Isl1-Cre; Rosa RFP^* cells with End2 cells greatly induced GFP^+^ (∼15%) or RFP^+^ (∼20%) cells from day 5, respectively (Figure 3E, F). Contrarily, the GFP^+^ or RFP^+^ cells were barely induced without End2 cells (Figure 3E, F). Similarly, the co-aggregation resulted in a marked induction of YFP^+^ cells from iPS cells harboring *Isl1^Cre^; Rosa^YFP^* (iPS*^Isl1-Cre; Rosa-YFP^* cells) [11] (Figure 3G).

To confirm the cardiogenic potential of End2-induced CPCs, ES*^Isl1-Cre; Rosa RFP^* cell-derived RFP^+^ cells were FACS-isolated after 6 days of co-culture with End2 cells and differentiated as a monolayer for 4 additional days in serum-free medium. Undifferentiated RFP^+^ cells showed high-level expression of CPC genes including *Isl1*, *Nkx2.5*, *Gata4* and *Tbx5* (Figure 3H). After 4 days of differentiation, these cells formed a sheet of beating cardiomyocytes demonstrating their cardiogenic potential (Movie S1). They were also positive for the cardiomyocyte marker cardiac troponin T, the smooth muscle marker alpha smooth muscle actin or the fibroblast marker vimentin (Figure 3I, J). Consistently, qPCR analyses revealed that these cells expressed high levels of cardiac transcription factors (*Nkx2.5*, *Gata4*, *Tbx5*), cardiomyocyte markers (*Actc1*, *Tnnt2*, *Myl2*) and smooth muscle markers (*Tagln*, *Vimentin*). The differentiated RFP^+^ cells did not express the pluripotency marker *Pou5f1* or the other germlayer markers *Afp* (endodermal) and *Sox1* (ectodermal) (Figure 3H). Taken together, these data suggest that co-aggregation with End2 cells efficiently induces committed CPCs from mES/iPS cells.

### Co-Aggregation with End2 Cells Promote Induction of Cardiac Progenitors from Human Embryonic Stem Cells

**Figure 4:**
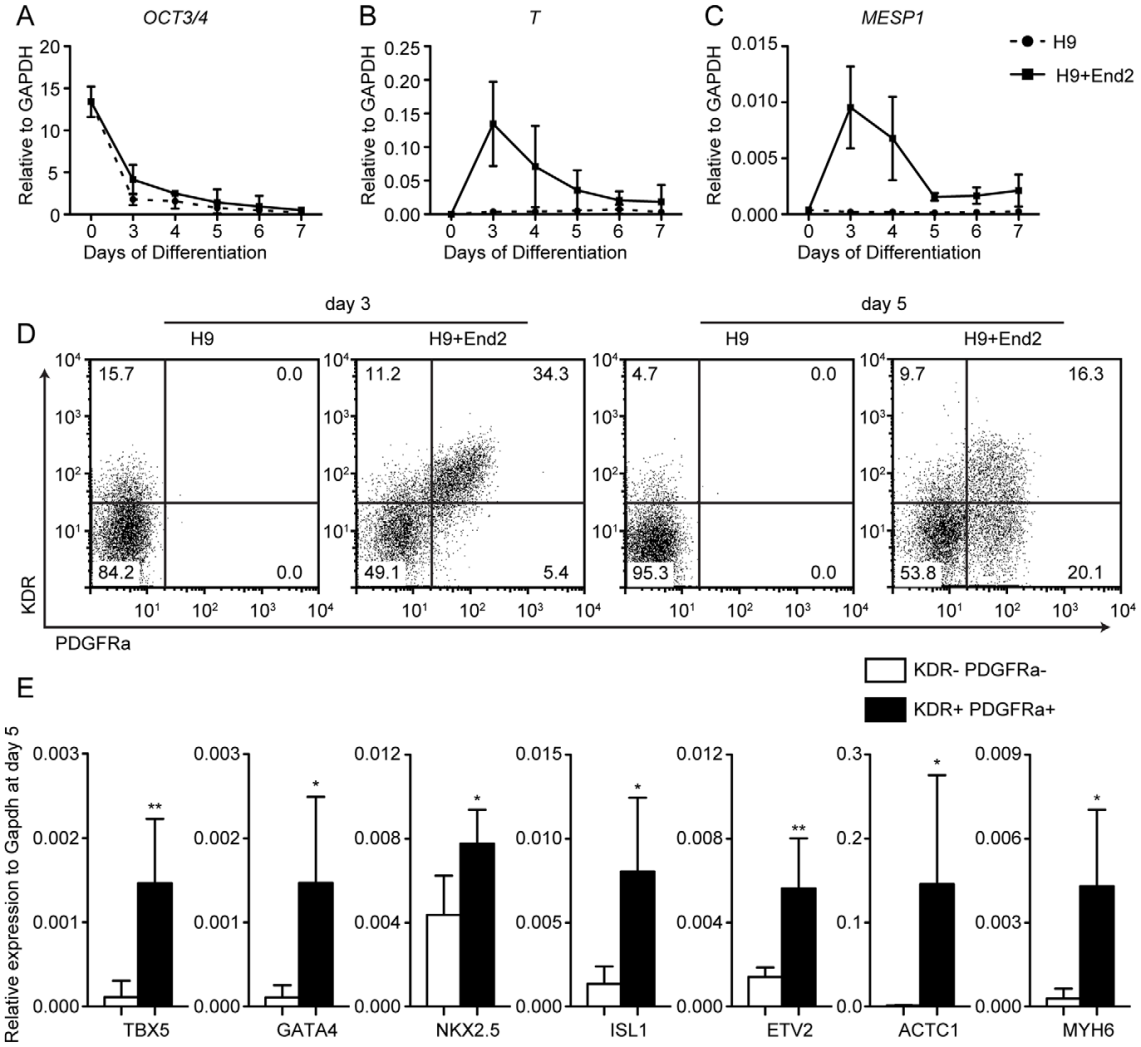
Co-aggregation with End2 cells efficiently induces cardiac progenitor cells from human ES cells. **A–C**, Gene expression profiles during human ES cells differentiation with or without End2 (n = 4, mean±SD). Undifferentiated cell marker, *OCT3/4* (A), mesendoderm marker, *T* (B), pre-cardiac mesoderm marker, *MESP1* (C). **D**, Plots of pre-cardiac mesoderm. KDR^+^ PDGFRa^+^ cells were only observed in EBs with End2 cells. **E**, Gene signature of KDR^+^ PDGFRa^+^ cells and KDR^−^ PDGFRa^−^ cells at day 5 of differentiation. Cardiac progenitor markers (*TBX5*, *GATA4*, *NKX2.5* and *ISL1*) and vascular progenitor marker (*ETV2*) were significantly upregulated in KDR^+^ PDGFRa^+^ cells. Early cardiomyocyte markers (*ACTC1* and *MYH6*) were also significantly upregulated. (n = 5, mean + SD, *; *p* < 0.05, **; *p* < 0.01, unpaired *t*-test).

To determine if End2 cells also induce pre-cardiac mesodermal cells from human pluripotent cells, we differentiated H9 hES cells by co-aggregation with End2 cells in suspension for up to 7 days and checked gene expression. The undifferentiated ES cell marker, *OCT3/4,* was rapidly downregulated with or without End2 cells (Figure 4A), indicating differentiation of hES cells in both conditions. However, only End2-aggregated hES cells exhibited a marked upregulation of *T* and *MESP1* (Figure 4B and 4C). Consistently, the number of KDR^+^ PDGFRa^+^ cells were greatly increased in hES cells with End2 cells from day 3 (Figure 4D), indicating that End2 cells promotes the induction of pre-cardiac mesodermal cells from hES cells. By day 5, the hES cell-derived KDR^+^ PDGFRa^+^ cells expressed high levels of genes marking committed CPCs (*TBX5*, *GATA4*, *NKX2.5*, *ISL1*), vascular progenitors (*ETV2*) and immature myocytes (*ACTC1*, *MYH6*) (Figure 4E). Endothelial marker (*PECAM1*) and relatively mature myocyte marker (*MYH7*) were not detected yet in CPCs (Data not shown). Together, these data suggest that co-aggregation with End2 cells potently induces CPCs from hES cells, analogous to the induction from mES cells.

## Discussion

To date, a number of cardiac differentiation methods have been reported, including conventional serum-based methods, feeder cell methods, and methods using defined culture media with specific cytokines or small molecules [7], [8], [22], [23]. Cytokine-independent methods are simple, but not suitable for CPC research due to their poor efficiency in CPC induction. Cytokine-dependent methods could be efficient, but are not widely used because they typically have multiple steps, which are highly sensitive to timing and cytokine levels, and require extensive and cell line-specific optimization [9], [10]. Moreover, cytokines are expensive and often show lot-to-lot inconsistency.

End2 cells were reported to promote cardiomyogenesis from hES cells [13]. However, the earlier studies focused on cardiomyocyte differentiation as a readout, and it was completely unknown if End2 cells affect CPC development. Based on the in-vivo process that endodermal cells physically contact with nascent CPCs until the cardiac crescent stage, we set out to test if direct contact with endodermal cells affects CPC induction. With our extensive analysis using cell surface markers and specific CPC reporter cell lines, we demonstrated that End2 cells can enhance CPC induction through cell-cell contact. Although End2 cells are known to secrete factors involved in cardiac differentiation (ActivinA/TGFβ, BMPs and Prostacyclins), [24], [25] the levels may not sufficient to induce CPCs as End2-conditioned media has no affect on CPC induction.

The present work provides a novel method for CPC induction in mouse and human pluripotent cell systems. We demonstrated that early endoderm maintains close contact with nascent CPCs in vivo and co-aggregation with the endoderm-like End2 cells potently induces CPCs in vitro. The method offers a simple, efficient and cost-effective way to induce committed CPCs from mouse and human pluripotent cells. We believe our new method will greatly benefit current CPC research and accelerate discovery of future CPC-mediated cardiac therapeutics.

## Supporting Information

Movie S1 **Spontaneously contracting sheet of cardiomyocytes differentiated from End2-induced CPCs.**
(MOV)
